# Utilizing Deep Learning for Diagnosing Radicular Cysts

**DOI:** 10.3390/diagnostics14131443

**Published:** 2024-07-06

**Authors:** Mario Rašić, Mario Tropčić, Jure Pupić-Bakrač, Marko Subašić, Igor Čvrljević, Emil Dediol

**Affiliations:** 1 Clinic for Tumors, Clinical Hospital Center “Sisters of Mercy”, Ilica 197, 10000 Zagreb, Croatia; 2 Faculty of Electrical Engineering and Computing, University of Zagreb, Unska ulica 3, 10000 Zagreb, Croatia; mario.tropcic@fer.hr (M.T.); marko.subas@fer.hr (M.S.); 3 Department of Otorhinolaryngology and Maxillofacial Surgery, General Hospital Zadar, 23000 Zadar, Croatia; jureppbkr2@gmail.com; 4 Department of Maxillofacial and Oral Surgery, Dubrava University Hospital, Avenija Gojka Šuška 6, 10000 Zagreb, Croatia; igor.cvrljevic@gmail.com

**Keywords:** deep learning, panoramic radiography, radicular cysts, artificial intelligence

## Abstract

Objectives: The purpose of this study was to develop a deep learning algorithm capable of diagnosing radicular cysts in the lower jaw on panoramic radiographs. Materials and Methods: In this study, we conducted a comprehensive analysis of 138 radicular cysts and 100 normal panoramic radiographs collected from 2013 to 2023 at Clinical Hospital Dubrava. The images were annotated by a team comprising a radiologist and a maxillofacial surgeon, utilizing the GNU Image Manipulation Program. Furthermore, the dataset was enriched through the application of various augmentation techniques to improve its robustness. The evaluation of the algorithm’s performance and a deep dive into its mechanics were achieved using performance metrics and EigenCAM maps. Results: In the task of diagnosing radicular cysts, the initial algorithm performance—without the use of augmentation techniques—yielded the following scores: precision at 85.8%, recall at 66.7%, mean average precision (mAP)@50 threshold at 70.9%, and mAP@50-95 thresholds at 60.2%. The introduction of image augmentation techniques led to the precision of 74%, recall of 77.8%, mAP@50 threshold to 89.6%, and mAP@50-95 thresholds of 71.7, respectively. Also, the precision and recall were transformed into F1 scores to provide a balanced evaluation of model performance. The weighted function of these metrics determined the overall efficacy of our models. In our evaluation, non-augmented data achieved F1 scores of 0.750, while augmented data achieved slightly higher scores of 0.758. Conclusion: Our study underscores the pivotal role that deep learning is poised to play in the future of oral and maxillofacial radiology. Furthermore, the algorithm developed through this research demonstrates a capability to diagnose radicular cysts accurately, heralding a significant advancement in the field.

## 1. Introduction

A radicular cyst, also known as a periapical cyst, is the most common jaw cyst and represents one of the complications of dental caries. The development of radicular cysts is primarily associated with chronic periapical inflammation resulting from dental caries, although their formation can also be triggered by dental trauma or inadequate root canal treatment [1,2]. Radicular cysts are usually asymptomatic and are often discovered incidentally during routine radiological examinations. Many patients with a radicular cyst do not show symptoms until the cyst reaches a significant size or secondary infection occurs. Symptoms may include mild to moderate pain, swelling in the area of the affected tooth, and sometimes a visible change in the color of the tooth. Additionally, the causative tooth is often sensitive to percussion and usually tests negative for sensitivity [3]. A radicular cyst is typically a solitary lesion that can develop in both the upper and lower jaws, more commonly occurring in the upper jaw. This is likely due to the increased incidence of pulpal damage to the anterior maxillary teeth from trauma or palatal invaginations. In the lower jaw, they are most frequently located in the molar and premolar regions. Diagnostically, radicular cysts are most often identified on radiographs as radiolucent lesions surrounding the tooth root, often encased by a dense rim of cortical bone. Common radiographic techniques include panoramic radiographs, periapical radiographs, and cone beam computed tomography (CBCT) [4].

Treatment of radicular cysts involves surgical removal, which can range from enucleation to marsupialization, depending on the size and location of the cyst. Marsupialization is particularly significant for large cysts as it allows for the reduction in lesion size, facilitating eventual enucleation or healing. Recent case studies highlight the successful management of large radicular cysts through marsupialization, followed by orthodontic treatment to correct tooth displacement. Additionally, new perspectives suggest that some radicular cysts may heal after conventional root canal therapy, emphasizing the importance of conservative treatment approaches in certain cases [5,6,7].

Deep learning represents a specialized discipline within machine learning, focused on solving problems in the field of AI. Deep learning techniques have revolutionized the way computers recognize patterns and make sense of complex, unstructured data, enabling advancements in various areas such as speech recognition, visual object recognition, autonomous vehicles, medical diagnostics, and many others. Its ability to extract complex patterns from large datasets makes it ideal for solving problems that were unattainable with traditional algorithms. Deep learning employs algorithms inspired by the structure and function of the human brain, known as artificial neural networks [8,9]. These networks consist of layers of nodes or “neurons”, where each layer performs specific computations on the input data. The data pass through the layers, with each layer extracting and enhancing features relevant to the task at hand [10].

Panoramic radiographs depict the bone structures and teeth of the upper and lower jaw, facilitating the assessment of the size and position of the cyst relative to other anatomical elements. The imaging process is simple and non-invasive and can even be performed on patients unable to open their mouths. Additionally, panoramic radiography is relatively cost-effective and easily available, making it an ideal choice as the first-line diagnostic tool in cases of suspected cystic lesions of the lower jaw.

While this method offers numerous advantages, there are certain limitations to consider. Panoramic radiography does not provide the same level of detail as CT scans, especially in more complex situations. Overlapping structures can occur, making it difficult to identify smaller cysts or lesions in the early stages of development [11,12].

Since it is a two-dimensional representation, accurately determining depth and precise spatial relationships can be challenging. Therefore, proper interpretation of panoramic images requires the experience and expertise of dentists, radiologists, or surgeons [13].

Panoramic radiographs serve as a cornerstone in the field of dental radiology, providing a unique and comprehensive view of the oral cavity and playing a crucial role in the diagnosis, treatment planning, and monitoring of various dental and maxillofacial conditions, despite certain limitations.

AI is increasingly being integrated into oral and maxillofacial surgery, offering significant improvements in diagnostics, treatment planning, and the execution of surgical operations. AI can quickly and accurately interpret panoramic radiographs, identifying anomalies such as tumors, fractures, and other pathologies with high precision.

Chattopadhyay et al. developed a computer vision algorithm that can recognize occlusion disorders of the upper and lower jaws with an accuracy of 81%, while Xi et al. used automatic segmentation to determine bone volume and density [14,15].

Xiong et al., in their pilot study, created a deep neural network model for detecting caries based on intraoral images. They tested the model’s performance against a dentist with one year of professional experience. The developed model diagnosed caries at a higher rate than the clinician [16]. Ma et al. successfully developed an algorithm for detecting dental pulp inflammation using a convolutional neural network. The sample consisted of 348 intraoral periapical images, and the algorithm’s accuracy was 85% [17].

YOU ONLY LOOK ONCE (YOLO) refers to an object detection algorithm that represents a pivotal breakthrough in the field of computer vision technology. This algorithm has set new standards in efficiency and accuracy, surpassing the performance of renowned methods such as R-CNN, Fast R-CNN, Faster R-CNN, and SSD [18,19,20]. Since its initial introduction in 2016 with YOLOv1, YOLO models have undergone several upgrades and new versions, leading up to the latest iteration, YOLOv8, developed by the Ultralytics team. Each version has brought improvements in speed, accuracy, and model architecture, significantly contributing to object detection. YOLOv8 has introduced significant upgrades compared to previous models and utilizes an anchor-free approach. In other words, the model predicts the object’s position directly, without any adjustments, from a predefined anchor box, simplifying the detection process and reducing the number of required predictions. Changes have been made to the convolutional blocks of the model, where the first 6 × 6 convolution is replaced with 3 × 3, and mosaic augmentation is employed during training, where four different images from the dataset are merged into one image, with each of the original images occupying one quadrant of the new image. An alternative to the YOLO v8 series could be other frameworks such as EfficientDet, Faster R-CNN, or SSD (Single Shot MultiBox Detector). YOlov8 has been favored over alternatives primarily due to several key advantages. YOLO processes images much faster than many other object detection models, achieving real-time performance by processing frames in milliseconds. Unlike some alternatives that involve multiple stages or region proposals, YOLO utilizes a single neural network to directly predict bounding boxes and class probabilities from full images. This streamlined approach simplifies the pipeline and enhances efficiency. Moreover, YOLO models are open source and benefit from robust community support, which facilitates easier adoption and customization. Its architecture is adaptable and can be optimized for various hardware platforms and deployment scenarios, including edge devices, GPUs, and specialized hardware like TPUs (Tensor Processing Units). This flexibility enables efficient deployment in real-time applications where quick decision-making based on visual data is crucial, such as in robotics, surveillance, and medical imaging [21,22].

The purpose of this study was to diagnose radicular cysts in the lower jaw by developing a deep learning model based on a real-time object detection system, YOLOv8. Furthermore, the aim of this research was to explore and validate the potential applications of deep learning and AI in maxillofacial surgery.

## 2. Materials and Methods

This study was approved by the ethics committee of Clinical Hospital Dubrava (2023/2103-01) and performed in accordance with the tenets of the Declaration of Helsinki.

### 2.1. Panoramic Radiographs Selection

Patients were searched within the Hospital Information System (HIS) of Clinical Hospital Dubrava and the Department of Oral Surgery from 2013 to 2023, using the diagnosis K04.8 according to the International Classification of Diseases (ICD). The inclusion criteria required the presence of a radicular cyst in the lower jaw, confirmed by an oral surgeon, a radiologist, and a maxillofacial surgeon, along with histopathological verification of the diagnosis.

**Figure 1:**
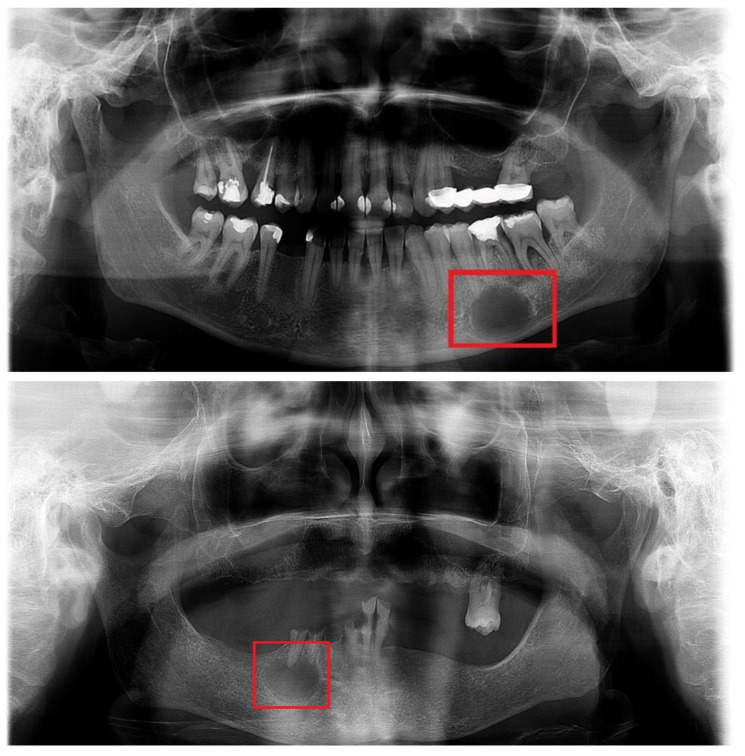
Examples of the included radicular cysts.

A total of 138 panoramic radiographs with radicular cysts were obtained, and an additional 100 panoramic radiographs excluding underlying medical conditions were included for comparative purposes (Figure 1). The digital panoramic radiographs were obtained using CRANEX 3D (Planmeca OY, Helsinki, Finland).

### 2.2. Preparation of the Imaging Datasets

The digital panoramic radiographs were carefully retrieved from the image database and saved in the Joint Photographic Experts Group (.JPEG) format, maintaining a resolution of 2776 × 1480 pixels. To develop an AI model that can label and diagnose radicular cysts of the lower jaw, the previously saved images needed to be prepared for annotation. Preparation was carried out by an oral surgeon, a radiologist, and a maxillofacial surgeon. The process involved marking the radiolucent lesion on each image using the GIMP program (GNU Image Manipulation Program). GIMP is free and open-source image editing software available for various operating systems, including Linux, macOS, and Windows. It was developed by a volunteer team of programmers through the GNU project and offers a rich set of tools for image processing, drawing, color correction, and format conversion. One of the key features of GIMP is the ability to import and save files in different formats, meeting the needs of a large number of users, and it is also open-source software.

**Figure 2:**
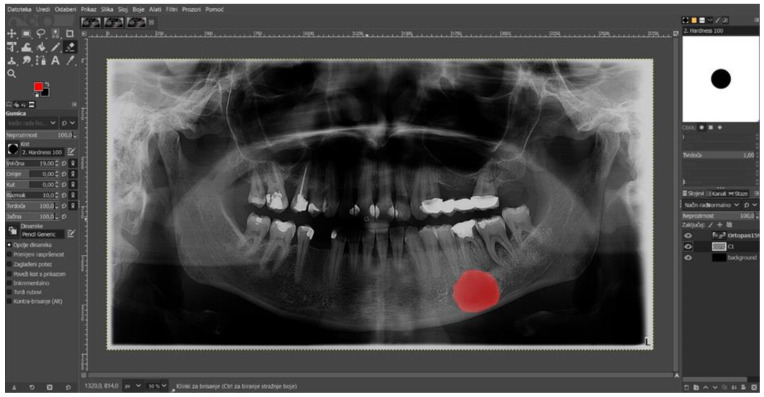
Manually annotated radicular cyst in the GIMP program located in the lower jaw.

The previously saved images in JPEG format were imported into GIMP 2.10.32 software. The image were then converted into a semi-transparent layer by changing the opacity parameter to 60 percent. This process makes the loaded image 60 percent visible and 40 percent transparent, which facilitates easier lesion marking. To ensure precise annotation, the brush settings used to draw the lesion edges need to be adjusted. In this study, a circular-shaped brush with 100 percent hardness was used. After completing all of the previous steps, the zoom-in option was selected for the lesion, and the lesion was marked with the brush. During the marking process, errors occurred in filling the lesion with the red brush, which were corrected using the paint bucket tool. The fully marked radiolucent lesion was saved in XCF (eXperimental Computing Facility) format for further preparation for AI model development (Figure 2).

### 2.3. Model

For this research, we chose to utilize the pretrained YOLOv8m and YOLOv8l models due to their advanced capabilities and proven effectiveness in various computer vision tasks. YOLOv8 stands out for its exceptional flexibility, capable of accurately identifying both singular and multiple object types depending on the training it undergoes. This model follows a highly efficient architecture, featuring a “backbone” for feature extraction and a “head” for predictions, ensuring robust feature extraction and precise predictions. The backbone of YOLOv8 employs a complex structure that includes multiple layers such as convolutional, batch normalization, and spatial pyramid pooling layers. These layers work together to extract and refine features throughout the network, capturing intricate details in the images. The head makes detailed predictions using these refined features, incorporating concatenation and upsampling techniques to enhance feature map resolution. These technical aspects contribute to YOLOv8’s ability to deliver high accuracy and fast processing speeds, making it ideal for real-time applications. YOLO v8m refers to a medium-sized version of the YOLO v8 architecture. It typically strikes a balance between speed and accuracy, making it suitable for applications where real-time processing is a priority but with moderate computational resources. YOLO v8l denotes a larger version of the YOLO v8 architecture. It is designed to prioritize accuracy over speed, making it suitable for applications where high precision in object detection is critical, even at the expense of slightly slower processing times and higher computational requirements.

We applied various augmentation techniques, including translation, scaling, horizontal flipping, rotation, and mosaic augmentation. These techniques were chosen to reflect natural variations in image capture, enhancing the model’s generalization capability. Despite appearing minor to the human eye, these transformations are perceived as new data by the model, significantly boosting its performance. Mosaic augmentation, in particular, has demonstrated outstanding results in numerous computer vision applications. The configuration file manages these augmentations, specifying the probability of applying mosaic and horizontal flipping, and setting the ranges for translation, scaling, and rotation. Augmentation was applied during each training epoch, ensuring that the model was exposed to a rich and varied dataset.

We configured the training process similarly to our previous research, with 100 epochs and a batch size of 4, constrained by the memory limits of our hardware and the high resolution of our images (2776 × 1480). Stochastic gradient descent was selected as the optimization algorithm, and training was executed on an NVIDIA RTX A6000 GPU to ensure rapid processing. To maintain consistent evaluation metrics on the test set, all training sessions were conducted deterministically. The dataset was divided into subsets according to the ratio of 3:1:1, with a larger portion for training and smaller portions for testing and validation.

### 2.4. Eigen Camera

EigenCam, short for Eigen Camera, refers to a technique used in computer vision and image processing to highlight the regions of an image that contribute most significantly to the predictions made by a deep learning model, particularly convolutional neural networks (CNNs). This technique is based on the concept of eigenvalues and eigenvectors from linear algebra, which help identify the principal components or the most significant features in a dataset. EigenCam provides insights into what the model is focusing on when making a prediction, which helps in understanding and interpreting the model’s decisions. This is particularly important in fields like medical imaging, where understanding why a model made a particular diagnosis is crucial.

By visualizing the important regions, we identified whether the model was focusing on the correct parts of the image or if it was being misled by irrelevant features. This aids in improving the model by refining the training data or the model architecture.

### 2.5. Metric Analysis

To examine the diagnostic accuracy of the AI model, metric analyses of precision, recall, and mean average precision were employed.

Mathematically, precision can be defined as the ratio of true positive predictions (TP) to the sum of true positive and false positive predictions (TP + FP), where:

TP (True Positives): The number of examples correctly classified as positive.

FP (False Positives): The number of examples incorrectly classified as positive.

The mathematical formula is:

(1) $$P=\frac{TP}{TP+FP}$$

High precision indicates that the model effectively avoids false positive errors, meaning that when the model classifies something as positive, there is a high probability that it is correct. However, precision does not consider all true positive examples (including those the model has failed to identify), which can be a limitation. Hence, we also used the metric of mean average precision and recall to obtain a more comprehensive view of the model’s performance.

Recall shows what portion of actual positive examples the model successfully identified as positive. Mathematically, recall is defined as the ratio of true positive predictions (TP) to the sum of true positive and false negative predictions (TP + FN), where:

TP (True Positives): The number of examples correctly classified as positive.

FN (False Negatives): The number of examples incorrectly classified as negative, though they are actually positive.

The formula for calculating recall is:

(2) $$R=\frac{TP}{TP+FN}$$

High recall indicates that the model identifies actual positive examples well. Additionally, to demonstrate the model’s success, mean average precision (mAP) and average precision (AP) were utilized.

mAP considers both precision and recall of the model, providing a comprehensive insight into the quality and accuracy of the model considering the entire set of predictions.

The mathematical formula is:

(3) $$mAP=\frac{1}{C}\sum_{n=1}^{c}AP$$

C is the number of classes or queries and AP is the average precision for the class or query. In the context of object detection, mAP can be computed at different intersection over union (IoU) thresholds. IoU is a measure of overlap between the predicted bounding box and the ground truth bounding box. For example, mAP@50 refers to mAP calculated at an IoU threshold of 50.

Additionally, the F1 score was utilized to measure the harmonic mean of precision and recall. It balances the precision and recall of a model and is especially useful when the class distribution is imbalanced.

The mathematical formula is:

(4) $$F1\;score=2\times \frac{Precision\times Recall}{Precision+Recall}$$

Data were collected and stored in a database in MS Excel.

## 3. Results

**Figure 3:**
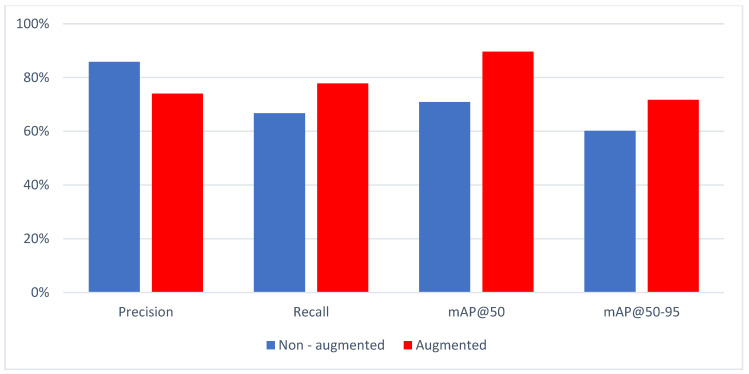
Metrical analysis of non-augmented and augmented data.

The test set performance of the trained model is presented in Table 1 and Figure 3.

Precision and recall were transformed into F1 scores to provide a balanced evaluation of model performance. The weighted function of these metrics determined the overall efficacy of our models. In our evaluation, non-augmented data achieved F1 scores of 0.750, while augmented data achieved slightly higher scores of 0.758. Additionally, our models achieved mAP@50 scores of 0.709 and 0.896 for non-augmented and augmented data, respectively. Furthermore, the mAP@50-95 scores were 0.602 and 0.717 for non-augmented and augmented data, respectively. Despite the lower precision observed when trained on augmented data, the improved F1 scores suggest an overall enhancement in detection performance.

These results underscore the significance of considering both precision, recall, and mAP scores in evaluating model performance, especially in medical imaging tasks like cyst diagnosis. The precision, recall, mA@P50, and mAP@50-95 scores without augmentation were recorded at 85.8%, 66.7%, 70.9%, and 60.2%, respectively. However, with augmentation, precision, and recall, mA@P50 and mA@P50-95 were 74%, 77.8%, 89.6%, and 71.7%, respectively.

**Figure 4:**
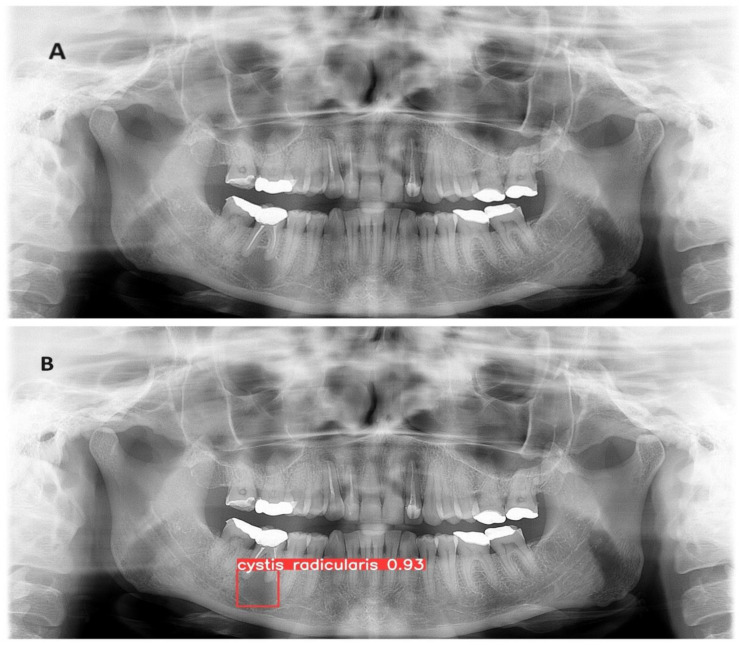
(**A**) Panoramic radiograph of radicular cyst in the lower jaw (**B**) Developed model detection prediction of a radicular cyst with precision of 93%.

**Figure 5:**
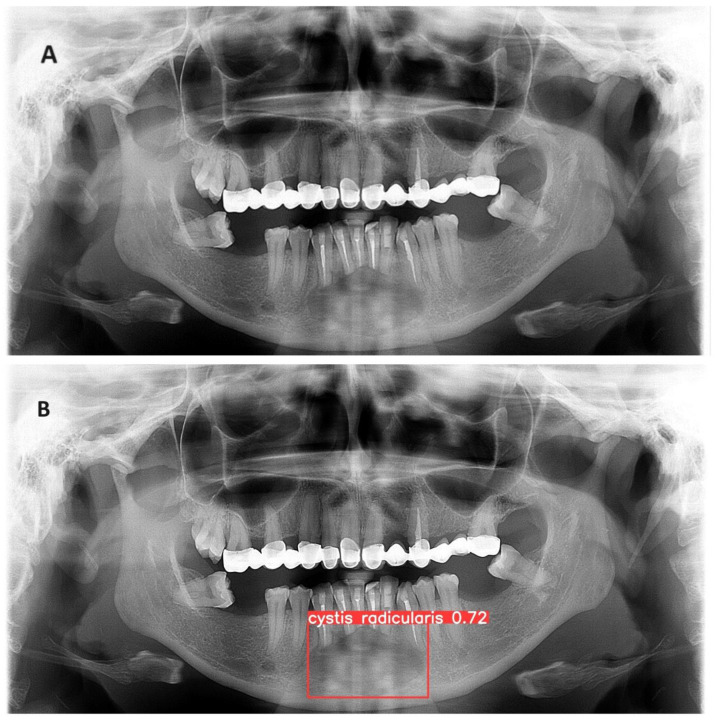
(**A**) Panoramic radiograph of radicular cyst in the lower jaw with surgically removed roots (**B**) Developed model detection of a radicular with precision of 72%.

The trained model is capable of predicting bounding boxes and probabilities as shown in Figure 4 and Figure 5.

In addition to evaluating the model’s performance on cases with cysts, we also conducted tests on a separate set of 100 orthopantomograms devoid of lesions and excluding any other underlying medical issues. Remarkably, the model demonstrated impeccable performance on this set, yielding no false positives, affirming its robustness in accurately identifying the absence of anomalies in such cases.

**Figure 6:**
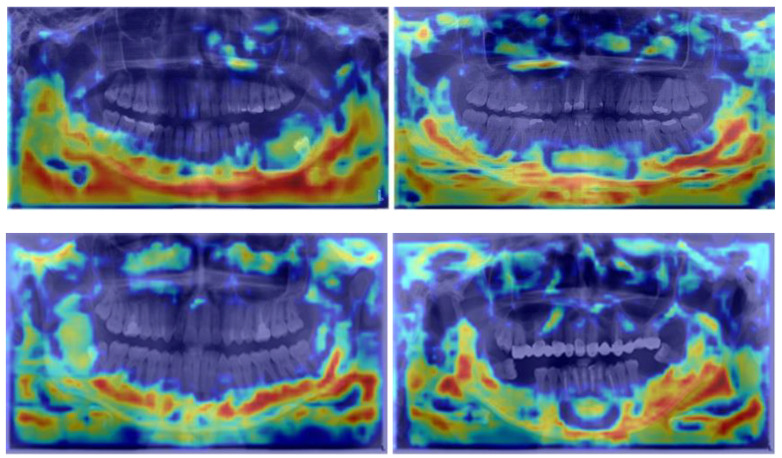
An intensified thermal zone is visible in the lower jaw area.

Furthermore, EigenCam was employed to gain deeper insights into the model, as depicted in Figure 6. In thermal imaging, each pixel represents a temperature value. An EigenCAM thermal zone would therefore highlight the areas with temperature variations that are most relevant to the model’s predictions. Additionally, there are some cold zones in the maxillary sinus, indicating that our algorithm faces challenges in distinguishing between the sinus and cysts. The red thermal zone in the lower jaw demonstrates higher accuracy, particularly around the dental roots. The concentration of intensity on the thermal map around the area of the lower jaw confirms the previous assertion and highlights the model’s capability to discern features pertinent to detecting mandibular cysts.

## 4. Discussion

The existence of radiolucent lesions in the lower jaw presents complex challenges in clinical diagnosis and treatment due to their varied morphology and potential complications. Consequently, the development of automated tools for precise detection, accurate segmentation, and reliable diagnostic classification holds significant potential in advancing patient care and treatment outcomes in the field of oral radiology and maxillofacial surgery.

AI has found applications in various fields, including medicine and dentistry. As early as the 1970s, the MYCIN system for diagnosing bacterial infections was developed, significant due to its ability to draw conclusions based on uncertain or incomplete information, using rules grounded in expert knowledge from the medical field. Functioning through questionnaires filled out by physicians about patients and 600 rules for differentially diagnosing various bacterial infections and recommending antibiotics [23,24,25]. Leveraging the MYCIN platform, Henry Pople developed the INTERNIST-1 medical diagnostic algorithm, later upgraded to the CADUCEUS expert system. CADUCEUS, based on the knowledge of American molecular biologist John Edgar Myers, could diagnose over a thousand different diseases. Dxplain, developed at the University of Massachusetts, provided students and physicians insight into a list of differential diagnoses for over five thousand different diseases based on complex symptoms [26,27].

Esteva et al. trained a neural network to recognize and differentiate melanoma from other skin lesions, achieving the ability to distinguish at a level comparable to experienced dermatologists [28]. Gargeya et al. developed an impressive algorithm for diabetic retinopathy detection, achieving sensitivity and specificity of 94% and 98%, respectively [29].

Notably, Kim et al. from Yonsei University in South Korea developed an algorithm for myocardial infarction screening based on deep neural networks and a database of over 2000 participants, demonstrating impressive metric results including precision, recall, and F1-score of 99.38% [30].

Moreover, AI has been used in diagnosing Alzheimer’s disease by analyzing amyloid imaging databases, accurately detecting response to therapy. The significant presence of input data in these studies, used for developing UI models, is also a potential challenge [31].

Given the extensive input data in these studies, developing AI models, there is potential for issues such as data imbalance. For instance, most Western countries compile databases and employ personnel in healthcare institutions and universities solely dedicated to data entry, enabling them to have tens of thousands of input data for developing algorithms using neural networks.

Diagnosing and treating periapical lesions can be challenging for clinicians. The characteristics of periapical radiolucencies and alveolar bone resorption allow for the development of various UI algorithms. Endras et al. developed a model for diagnosing periapical lesions and tested it against maxillofacial surgeons, showing better diagnostic results compared to 14 out of 24 participating surgeons [32].

Ryong Ha et al. attempted, through machine learning, to recognize factors influencing the prognosis of osseointegration and the success of dental implant placement, finding that implants placed mesiodistally had the greatest impact on the final success of dental rehabilitation [33]. Additionally, in forensic dentistry, there is increasing use of deep neural networks in analyzing bite marks and estimating age and gender based on permanent dentition.

In light of AI’s wide application within the pathology of the oral and maxillofacial sphere, it is imperative to initiate research projects aimed at integrating such technological solutions into a coherent clinical decision support system.

In this study, a model for diagnosing radicular cysts of the lower jaw was developed using the most advanced version of YOLOv8. In our previous work, we utilized YOLOv8 for lesion segmentation and detection while in this study, we extended its application to the diagnosis of radicular cysts. As stated, according to the available literature, YOLOv8 has not been used for this purpose [34]. Kwon et al. used YOLOv3 in their study and utilized a dataset of 1282 radiolucent lesions. The developed model’s accuracy in detecting radiolucent lesions of the lower jaw was 87%, with a sensitivity of 83% [35]. Despite having a much larger dataset, their detection metrics were similar to ours. The precision in diagnosing radicular cyst of the lower jaw in our model was 74%, with an mAP50 of 89.6% and sensitivity of 77.8% (Table 1).

Additionally, Ariji et al. used the Detect-Net deep neural network in an attempt to develop a UI model for detecting jaw lesions, achieving a detection accuracy of 81% for radicular cysts [36]. In our study, the mAP50 with augmentation was 89.6% (Table 1).

Van Berne et al. utilized YOLOv3 and MobileNetV2 to develop an algorithm for classifying radicular cysts and periapical granulomas. Their final dataset comprised 80 radicular cysts and 72 periapical granulomas, with images resized to a resolution of 256 × 256 pixels. The model achieved a sensitivity of 1.00 (95% CI 0.63–1.00) for radicular cysts, a specificity of 0.95 (95% CI 0.86–0.99), and an AUC of 0.97 [37]. The images in our study had a resolution of 2776 × 1480 pixels.

Considering the relatively small amount of data in this study, a limitation is noted, although it is consistent with similar research.

Innovative visualization techniques, such as EigenCAM, further enrich our understanding of how models make predictions. EigenCAM generates heat maps that visually represent regions the model deems crucial for its predictions. These maps offer a clear visual insight into parts of the image critical for recognizing pathological conditions, often highlighting specific regions, such as the lower jaw, with heightened intensity, emphasizing potential locations of radiolucent lesions (Figure 6).

The findings of this study underscore the remarkable diagnostic proficiency of the developed model in identifying radicular cysts located in the lower jaw. A limitation of this study is the relatively small amount of input data used for model development and testing, though it is in line with similar studies. Implementing AI algorithms in medical practice presents numerous ethical considerations and challenges. AI systems depend on extensive patient data, which raises concerns about privacy breaches and the security of confidential medical information. Moreover, ensuring the accuracy, reliability, and safety of AI algorithms in medical settings necessitates rigorous clinical validation. Regulatory frameworks must evolve to effectively oversee the integration of AI in healthcare. Furthermore, AI’s involvement in medical decision-making has the potential to reshape the traditional physician–patient relationship, potentially diminishing direct interaction and human judgment. Access to AI-driven medical technologies may also widen existing disparities in healthcare access, particularly affecting underserved or marginalized communities.

Furthermore, it is essential to highlight that our research was limited to radicular cysts of the lower jaw. The algorithm encountered difficulties with the maxillary sinus when detecting radicular cysts in the upper jaw. Future studies could address this issue by expanding the application of YOLOv8 to include the upper jaw and different types of cysts, thereby enhancing its potential utility. The plan is to expand the dataset and adapt the algorithm for use in the upper jaw. Generalizing the algorithm is crucial to ensuring its effectiveness and reliability in clinical applications. Future research will focus on collecting panoramic radiographs from various sources and demographic groups, as well as adapting the algorithm based on feedback from clinical practice. Additionally, continuous testing on new, unseen data is planned to ensure that the algorithm maintains a high level of accuracy even in novel situations.

## 5. Conclusions

Considering the limiting factors of this study, it is concluded that artificial intelligence represents a powerful tool capable of detailed analysis of medical images. Additionally, the developed deep learning algorithm is capable of diagnosing radicular cysts of the lower jaw with impressive metric indicators. By analyzing how the developed model processes data through visualization activations and heat maps, we pave the way for its further refinement.

## Figures and Tables

**Table 1:** Performance of the model in the detection of radicular cysts with and without augmentation: precision (positive predictive value), recall (sensitivity), and mean average precision.

	P	R	mAP@50	mAP@50-95
Without augmentation	0.858	0.667	0.709	0.602
With augmentation	0.74	0.778	0.896	0.717

## Data Availability

The raw data supporting the conclusions of this article will be made available by the authors on request.
